# Clinical relevance of feto-maternal microchimerism in (hematopoietic stem cell) transplantation

**DOI:** 10.1007/s00281-024-01028-3

**Published:** 2024-12-07

**Authors:** Anne Kruchen, Boris Fehse, Ingo Müller

**Affiliations:** 1 Division of Pediatric Stem Cell Transplantation and Immunology, Clinic of Pediatric Hematology and Oncology, University Medical Center Hamburg-Eppendorf, 20246 Hamburg, Germany; 2 Research Department Cell and Gene Therapy, Department of Stem Cell Transplantation, University Medical Center Hamburg-Eppendorf, 20246 Hamburg, Germany; 3 Research Institute Children’s Cancer Center Hamburg, 20246 Hamburg, Germany; 4 German Center for Child and Adolescent Health (DZKJ), Hamburg, Germany

**Keywords:** Feto-maternal immune tolerance, Hematopoietic stem cell transplantation, Microchimerism, Non-inherited maternal antigen

## Abstract

Toleration of a semi-allogeneic fetus in the mother’s uterus as well as tolerance after allogeneic hematopoietic stem cell transplantation (HSCT) appear to share some immunologic concepts. The existence of microchimeric cells, and the original idea of a bidirectional cell trafficking between mother and child during pregnancy have been known for decades. Today, origins and mechanisms of persistence of microchimeric cells are intensively being elucidated. Both, the translation of the phenomenon of feto-maternal immune tolerance to donor choice or prevention of graft-versus-host disease (GvHD) in HSCT, and the implications of microchimeric cells in and for HSCT are highly intriguing. Yet, differences in detection methods of microchimeric cells, as well as in transplantation protocols impede the comparison of larger cohorts, and limit potential clinical advice. Still, matching of non-inherited maternal antigens (NIMA), which are expressed on maternal microchimeric cells, demonstrated a strong association with decreased risk for the development of acute GvHD in the context of various transplantation strategies. Despite the fact that advances in graft manipulation and immunosuppression ameliorated the safety and outcome after HSCT, NIMA-matching retained a beneficial role in selection of sibling, child, or maternal donors, as well as for cord blood units. Recent findings indicate the existence of a microchimeric stem cell niche, in which only one dominant microchimeric cell population of only one semi-allogeneic origin persists at a time. This implies that studies regarding the impact of (maternal and fetal) microchimerism (MC) on clinical outcome of HSCT should combine analysis of NIMA and direct detection of microchimeric cells from donor and recipient on the verge of HSCT to be efficiently conclusive.

## First descriptions and analyses of (hematopoietic) fetal or maternal microchimeric cells

 The idea of exchange of fetal and maternal cells during pregnancy was postulated by Georg Schmorl already in the 19th century. His studies at that time were focused on the deciphering and characterization of preeclampsia. Rather serendipitously, he identified placental cells in the pulmonary arteries of women who had succumbed to preeclampsia [76]. While only hypothesized by Schmorl, it was later proven that a bidirectional exchange of maternal cells into the fetus and fetal cells into the mother occurs in every pregnancy. Fetal microchimeric cells were detected in maternal peripheral blood as early as gestational week six, and can be found in several maternal tissues including lung, spleen, liver, kidney, heart, and brain [23, 58, 80, 108, 143]. Maternal microchimeric cells were first reported in the 1960s, when maternal leukocytes were identified in umbilical cord blood [122]. First evidence of maternal cells in the fetal circulation can be observed starting from the second trimester [79]. During its development the fetal thymus is firstly entered by hematopoietic progenitor cells from the peripheral blood by gestational week 8–9, and the first mature T cells can be detected about a week later [55]. In that timeline, maternal cells can supposedly only enter the fetus after development of the fetal thymus [55, 71]. Like fetal cells in the mother, maternal cells have been observed in diverse fetal tissues including lung, spleen, liver, kidney, pancreas, skin, thyroids, and thymus [125, 130].

Since the first description of fetal microchimeric cells observed during autopsies, detection technologies have become more and more precise. Today, the term microchimerism (MC) depicts the presence of < 1% allogeneic cells within an organism [4, 34]. These cells originate from feto-maternal cell exchange during pregnancy, but also from donor cells following organ transplantation, blood transfusions or microtransplantation. Hence, detection methods must meet high standards regarding specificity and sensitivity: Specificity is required to reliably discriminate between mother and child, or host and allogeneic source, sensitivity to allow for detection of small, yet biologically relevant amounts of microchimeric cells, ruling out false-negative results. For the first decades, fluorescent in situ hybridization (FISH) analysis to detect XY cells within female tissues was the predominantly applied detection method until Y-specific polymerase chain reaction (PCR) detection methods supervened [56, 91]. Real-time PCR methods for monitoring of donor chimerism after hematopoietic stem cell transplantation (HSCT) later also paved the way for female microchimeric cell detection [1, 39]. Other high-sensitivity methods that have been employed to detect microchimerism comprise flow cytometry (FC) [27], digital PCR [126], and next-generation sequencing (NGS) [2]. Discrimination between host and allogeneic origin is achieved by disparities in sex (Y chromosome), variable numbers of tandem repeats (VNTR) or characterization of short tandem repeats (STR), insertion/deletion polymorphisms (InDels), single nucleotide polymorphisms (SNPs), or HLA mismatches (reviewed in [4, 127]).

Taken together, technical difficulties in detection of rare cell populations and false-negative results due to limitations of older methods heavily affected the results of the studies covered by this review. In addition, comparing quantitative measurements between different approaches is highly error prone.

## Origin, persistence and sanctuaries of fetal or maternal microchimeric cells

The fact that the exchange of fetal and maternal cells has been conserved in mammalian species lets one surmise that there might be a biological benefit [95]. To further elucidate potential advantages, it is mandatory to understand the origins and biology of microchimeric cells.

### Origins and biology of fetal or maternal microchimeric cells

Naturally occurring microchimerism has been described in different research fields for decades. However, characterization and classification of these cells is still under development since the afforded methods needed to become more precise. Yet, detection alone leaves no information on the biology of the cells. Today, single-cell analysis provides the most in-depth insights. For microchimeric cells in general, many different origins have been postulated including all three germ layers (reviewed by Stahlberg et al. [127]). We here focus on implications in and for HSCT, with particular regard to microchimeric cells in the peripheral blood, cord blood and bone marrow. In fact, newer high-sensitivity detection methods indicate that maternal microchimerism is the rule rather than the exemption. Whereas Kanold et al. detected maternal MC in 11% of cord blood (CB) samples after Cesarean sections, in 2017, Kanaan et al. found even 53% of tested CB probes MC-positive. [67, 69]. In the study by Kanold et al. maternal microchimeric cells were characterized as CD34 (a stem cell marker) and/or CD56 positive [69]. In the study by Kanaan et al. the maternal microchimeric cells were numerically most abundant in memory, followed by naïve T cell subsets, but also present among B cells, NK cells, and monocytes [67]. Detection and characterization of fetal cells in the maternal hematopoietic system can be performed much easier due to better access to and larger sample volumes. Again, fetal microchimeric cells are mainly reported to be of hematopoietic progenitor or stem cell origin [53, 101]. Analyses of hematopoietic cells is facilitated by the use of flow cytometry techniques [34], employing both cell-type and fetal/maternal specific markers for concurrent characterization. High expression of the respective receptor, C-C chemokine receptor type 2 (CCR2), was reported on a subset of fetal microchimeric cells in mice, co-expressing CD31, CD34, and CD11b identifying them as bone marrow derived epithelial progenitor cells. The CCL2/CCR2 axis was lately identified as major player in recruitment of fetal microchimeric cells in maternal wound healing [22]. The advent of very efficient sorting technologies (fluorescence-activated cell sorting, FACS and magnetic cell sorting, MACS) has subsequently opened the opportunity for single-cell analyses of microchimeric cells, e.g. by RNA sequencing. So far, the latter has been employed in the characterization of maternal microchimeric cells in whole mouse embryos. Maternal microchimeric cells could be FACS-sorted based on GFP expression [45]. Subsequent single-cell sorting and RNA sequencing revealed mainly immune-related cells, 25% terminally differentiated cells, and 8% proliferating/stem cells. There was a high variation in the maternal cell compositions between the individual mouse embryos. Yet, myeloid cells were identified in more than 80% of the embryos, and about half of the embryos contained maternal endothelial cells, slamf1-negative multipotent progenitor cells, and granulocytes [46].

### Persistence of fetal and maternal microchimeric cells

Once established, fetal microchimerism was demonstrated to persist up to decades after parturition [17]. Some studies reported a positive correlation between female fetal sex and the prevalence of fetal microchimeric cells shortly after parturition as well as years after birth [41, 86]. Nevertheless, studies on microchimerism have been and, due to easier and faster analyses, often still are being performed via the detection of Y-chromosome positive cells within a female host. This methodologic limitation not only leads to biased results but also neglects the long-term monitoring of individual childs in the mother.

Histocompatibility between fetus and mother has been proposed to be an important player for persistence of naturally transmitted microchimeric cells. Persistence of maternal cells in the offspring was described to be affected by human leukocyte antigen (HLA) class II compatibility, especially HLA-DRB1 and/or -DQB1 [14]. In mice, major histocompatibility complex (MHC) -matching also resulted in higher rates of maternal microchimeric cells in offspring [70]. Vice versa, the occurrence of fetal microchimeric cells within mothers was positively correlated with HLA compatibility. HLA-DQA1*0501 in mothers or sons was associated with fetal microchimerism [77]. Similar findings were made for HLA class I molecules: presence of an HLA-C1 allele, in particular an HLA-C match between mother and child favored persistence of fetal microchimeric cells [88].

Recently, Shao et al. revealed the displacement of fetal microchimeric cells from a first pregnancy by fetal microchimeric cells of subsequent pregnancies in a mouse model. Equally, maternal chimeric cells in daughters are exchanged by the daughter’s offspring’s fetal chimeric cells with her first pregnancy [119]. The authors postulate that there can only be one predominant microchimeric cell lineage in one individual. Translation of these results to humans was demonstrated in a small cohort study by Gamill et al.: The total amount of microchimeric cells did not change despite multiparity, while the concentration of maternal microchimeric cells decreased with increasing pregnancies [47]. Similarly, it was reported that the frequencies of male microchimerism in women originating either from a son, male twin or an older male sibling are not significantly different. Furthermore, there was no additive effect if a woman had both, a son and an older male sibling [65]. Another interesting study, though with a rather small cohort, showed that women suffering from recurrent miscarriages presented with a lower amount of maternal microchimeric cells before pregnancy compared to women without complications. From 14 women with recurrent miscarriages, 11 subjects later successfully delivered a child. Out of this group, four women had a detectable maternal microchimerism during or after pregnancy, one presented with both fetal and maternal microchimerism, and one had a detectable fetal microchimerism. Three of the 14 women endured further miscarriages, two women without detectable maternal microchimerism during pregnancy, and one without detectable fetal microchimerism (maternal not analyzed) [48]. These data suggest that a displacement of microchimeric cells is indispensable for successful pregnancies.

In a cohort of 118 normotensive term pregnancies the prevalence and quantity of fetal microchimeric cells decreased with increasing levels of placental growth factor (PlGF), while their prevalence but not quantity was positively correlated with soluble fms-like tyrosine kinase-1 (sFlt-1) [42]. A decrease in PIGF and increase in sFlt-1 are markers for placental dysfunction [93]. Presence of maternal microchimeric cells in cord blood samples was reported to be associated with two factors - the first being high concentrations of maternal Pregnancy-Associated-Protein-A in the first trimester, the second being the compatibility between fetal and maternal HLA-A and/or DR. Furthermore, there was a higher proportion of CD56^+^ NK cells in cord blood samples positive for maternal microchimeric cells compared to those that lacked maternal microchimeric cells [54].

The observations that microchimeric cells have a limited number and are replaced by subsequent pregnancies might explain why persistent microchimerism can be detected in some but not all mothers, if the analysis is performed on selected children only.

### Sanctuaries of fetal and maternal microchimeric cells

**Fig. 1:**
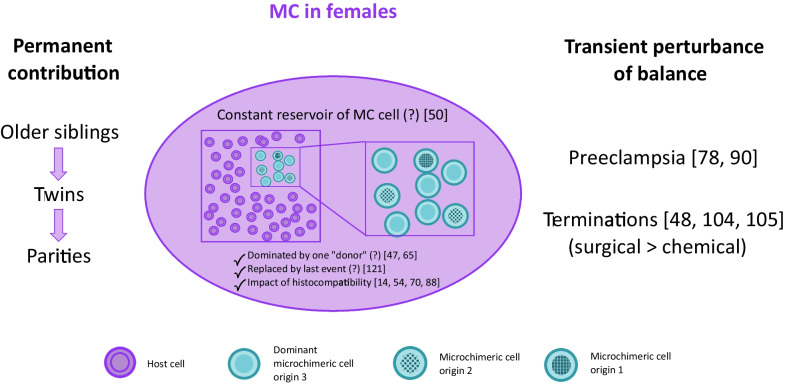
Dynamics of microchimeric cells in female hosts. The first microchimeric cells in a female individual are maternal and possibly of older sibling’s or a twin’s origin. With each pregnancy later on in life, these microchimeric cells will (in part) be exchanged with filial microchimeric cells. This balance might be perturbed by certain transient conditions like preeclampsia or surgical or chemical pregnancy terminations

As described above, the number of microchimeric cells within a female host seems to be restricted, and is not altered by multiparity (Fig. 1). Yet, it has been demonstrated, that certain circumstances and factors lead to a fluctuating emergence of microchimeric cells, some changes are probably only short term and/or only detectable in the peripheral circulation. In women diagnosed with preeclampsia the frequency of fetal microchimeric cells was higher in peripheral blood lymphocytes, primarily in B and NK cells, compared to stem cells circulating in peripheral blood [88]. A tremendous increase of fetal microchimeric cells was reported in maternal peripheral blood after the termination of pregnancies [103]. The level of microchimerism was also dependent on the procedure. Surgical terminations led to higher numbers of fetal microchimeric cells compared to chemical abortions [102, 103].

The importance of a certain histocompatibility for the establishment of feto-maternal microchimerism has been abundantly demonstrated [14, 35, 56, 77]. Yet, the cells are typically semi-allogeneic, and the question remains how especially maternal microchimeric cells survive in the offspring, before they are possibly exchanged by a new generation of fetal microchimeric cells during a first pregnancy. The mother more obviously needs to establish an immune tolerance to protect the child. Given the fact that hematopoietic stem cells only express low levels of HLA class I molecules and no HLA class II, microchimeric cells might preserve stem-cell like character, and as such populate sparse niches in the bone marrow. Indeed, in animal studies it was demonstrated that in more than 70% of the mice, lineage-negative maternal microchimeric cells could be detected in the bone marrow, whereas only half of the mice were positive for lineage-positive maternal microchimeric cells. Mesenchymal stromal cells (MSC) of maternal origin could also be isolated, though at low frequencies [33]. Strikingly, diverse sanctuaries for microchimeric stem cells have been reported [25]. Fetal-derived MSC, capable to differentiate into the osteogenic, chondrogenic and adipocyte lineages were successfully isolated from maternal hair follicles [26].

Microchimeric stem cells in their niche/sanctuary maintain the potential to become either beneficial or disadvantageous as soon as their progeny enters the peripheral circulation. A study analyzed the amounts and subsets of fetal microchimeric cells in women suffering from preeclampsia in comparison with healthy controls. Whereas fetal microchimeric stem cells were present at identical frequencies in the peripheral blood of both groups, numbers of fetal microchimeric lymphocytes were significantly higher in the patient group. It was hypothesized that these circulating immune cells are prone to become a trigger to chronic maternal disease, like Hashimoto’s thyroiditis, systemic lupus erythematosus, Sjögren’s syndrome, systemic sclerosis or primary biliary cirrhosis [43, 88].

## Immunological implications of MC for hematopoietic stem cell transplantation

For a broad overview on immunological implications of fetal or maternal microchimerism, we kindly refer to the review by Kinder et al. [71]. In HSCT, the selection of the best-suited donor is of crucial relevance. Depending on availability, HLA-matched related or unrelated donors are preferred. In case of absence or unavailability of the latter, HLA-haploidentical HSCT has become well established for treatment of high-risk leukemia in pediatric and adult patients [10, 72, 83, 85] and has been expanded to treat other hematologic and oncologic malignancies, including hemoglobinopathies and neuroblastoma [18, 44, 123]. Eligible HLA-haploidentical donors are usually either the biological mother or father, but can also be siblings or second-degree relatives. Hence, in most cases, the donor is immediately available and willing to donate stem cells as well as cells for potential later donor leukocyte infusions [96].

The discoveries of the major histocompatibility complex MHC comprising the histocompatibility genes and of the HLA molecules date back to the 1940ies and 1950ies, respectively [136]. Today, HLA incompatibility still remains a huge impediment, enhancing risks of life-threatening complications, such as graft rejection or graft-versus-host disease (GvHD). The incidence of GvHD increases with the degree of HLA disparity [133]. The major cause of GvHD are alloreactive T cells that can target mismatched HLA molecules either directly or indirectly and also recognize so-called minor histocompatibility antigens (minor H). At the same time, T-cell alloreactivity is most important for recognition and eradication of malignant cells underlying the graft-versus-leukemia effect (GvL). Notably, broadly expressed minor H antigens can induce GvHD and GvL likewise, while minor H antigens that are restricted to the hematopoietic system act predominantly beneficial in promoting GvL [6, 52, 124]. Aside from alloreactive T cells, HLA class I alleles are also ligands for killer inhibitory receptors (KIRs) on natural killer (NK) cells. NK cells are also potent inducers of GvL effects.

The relevance of MHC non-inherited maternal antigens (NIMA) in relation to minor H NIMA is highly challenging to address. Minor-H matched but MHC-mismatched HSCT in mice showed two different response models to NIMAs, high and low responders, depending on the level of maternal microchimerism. Low responders showed a better survival with less GvHD, accompanied by higher regulatory T cell (T_reg_) counts. Interestingly, these results were foreshadowed by less interferon (IFN)γ-producing cells upon stimulation with NIMAs before transplantation [5].

Before HSCT, recipient and potential donors are HLA-typed by DNA molecular analyses, and recipients are analyzed for preformed donor-specific anti-HLA antibodies which increase the risk of graft rejection and are most abundantly detected in parous women [51]. One would anticipate that anti-HLA antibodies are being produced by every mother against the paternally derived HLA on semi-allogeneic (in other terms haploidentical) fetal microchimeric cells. The existence of ´leukocyte antibodies´ and their clinical implications were already discovered by J. J. van Rood in the late 1950ies [140]. Yet, in earlier studies anti-paternal HLA antibodies could only be detected in around 20% of mothers. Furthermore, paternally derived HLA antigens had been described to appear masked in cord blood analyses. In serological analyses in the 1970ies only the maternally derived HLA could be typed effortlessly. Schroeder et al. described a correlation between the lack of fetal microchimeric cells in the mother and the detection of anti-HLA antibodies [117, 127]. Anti HLA-immunization could be detected in more than half of women after the first pregnancy and in three quarters of women after at least two pregnancies [87]. Once anti-HLA antibodies have been produced, memory B cells will have been established in turn, leading to a decade-long allo-immunization. Alongside anti-HLA antibodies, populations of anti-paternal HLA cytotoxic T cells (CTLs) were described, which can outlast antibody production [138, 139]. Together with anti-paternal HLA memory T cells, mothers might be faced with potential negative implications regarding donor compatibility. To balance out tolerance, a sufficient population of T_regs_ has to be present [105]. Allogeneic B-cell responses presented to be the major barrier in semi-allogeneic heart transplantations in mice. Whereas both fetus-specific T-cell tolerance and humoral sensitization were established simultaneously, the introduction of an imbalance by utilizing B-cell deficient mice led to markedly improved acceptance of the semi-allogeneic transplants postpartum [131]. Gillis-Buck and Gardner speculated that this concept could be adopted to human transplantation strategies. Women should hence be treated with an immunosuppression targeting pregnancy-induced antibodies, which does not affect the advantages brought about by pregnancy-induced T-cell tolerance [50].

### Pregnancy-induced T-cell tolerance

The immunologic protection of a semi-allogenic fetus is not yet fully elucidated. Indispensable herein is the role of CD4^+^ regulatory T cells (reviewed by Jiang et al. [64]). Human T_regs_ develop in the thymus and can be detected in thymic tissue from gestational week 13. Peripheral T_regs_ appear one week later, when human fetuses acquire T-cell immunity [28, 109]. In mothers, a systemic increase of T_regs_ in the peripheral blood is observed between the first and second trimester. Accordingly, in the decidua, at the maternal-fetal interface, maternal T_regs_ accumulate [57]. Around gestational week 14, human fetuses acquire T-cell immunity. Due to the different T-cell development in mice, mouse models can only give hints to human immunology. Transplantation of hematopoietic stem cells in fetal mice later than gestational day 14 faces higher grades of T-cell alloreactivity, resulting in lower engraftment rates. The combined infusion of host-derived T_regs_ with allogeneic HSC withstood alloreactivity from host T cells and resulted in no signs of GvHD [109].

In pregnant mice, T_regs_ can be detected from the day after conception and expand in successful pregnancies. The abundant presence of maternal T_regs_ is necessary to suppress anti-fetal immune responses. Indeed, the lack of functional T_regs_ might even lead to immunological rejection of the fetus [3, 113]. Fetus-specific T_regs_ are sustained after pregnancy and show an accelerated expansion in subsequent pregnancies. FoxP3^+^ T_regs_ are primed by fetal antigen stimulation, and their deficiency may result in fetal wastage [113, 119]. Sustained fetus-specific T_regs_ downregulate FoxP3 expression as well as surface expression of certain activation markers like CD25. This population of cells has been termed ‘ex-T_regs_’ [50]. Usually, T_regs_ are converted to ex-T_regs_ under inflammatory conditions, a mechanism which is not fully understood [115]. Ex-T_regs_ are maintained in the mother and will not be fully replaced by subsequent pregnancies [50, 119]. Vice versa, murine female offspring accumulates immune suppressive T_regs_ specific for NIMAs. When the mice are depleted from maternal microchimeric cells, the number of NIMA-specific T_regs_ is downregulated back to control levels. In other words, a certain level of maternal microchimeric cells apparently is required to maintain tolerance, and NIMA-tolerance is essential for reproductive advantages in pregnancies [70].

Post parturition, maternal cells might still migrate from mother to child via breast-feeding [94]. Alongside a possible contribution to maternal microchimerism, there was a higher abundance of T_regs_ in breastfed neonates compared to formula milk fed neonates [146]. This increased number was linked to tolerogenicity towards NIMAs accompanied by a decreased production of inflammatory cytokines [30, 146]. Altogether, breast-feeding seems to have a substantial influence on the level of microchimerism as well as on the establishment of a tolerogenic milieu.

Unimpeded maternal cell trafficking into a fetus lacking adequate immune responses can result in engraftment of large numbers of maternal T cells in the child and even lead to acute GvHD, as observed in neonates suffering from severe combined immunodeficiency (SCID) and thus completely lacking (functional) T cells [118]. Vice versa, a case of GvHD in a mother, three weeks after a complicated delivery involving hemorrhage, induced by fetal cells was recently also described [116]. On the other hand, maternal T cells can provide selective immunity and protect against early life infections [128]. CMV-specific CD8^+^ T cells of maternal origin were described in a young child suffering from X-linked SCID. Before and after haploidentical HSCT from the mother the cells exerted anti-CMV function in vitro [73]. In mice, maternal microchimeric cells have been reported to compensate either immunoglobulin G production in B-cell deficient offspring or interleukin-2 (IL-2) secretion in IL-2 deficient offspring [8, 147].

Whereas the (unhindered) cell trafficking mentioned above leads to rather prompt GvHD events, similar clinical events have been described in some autoimmune diseases. In autoimmune diseases in mothers, an increase in the amount of fetal cells can be demonstrated (as can usually in affected tissues in other diseases), and it has been proven that these fetal microchimeric cells are derived from circulating cells. The clinical symptoms of the described autoimmune diseases evoke the clinical symptoms of GvHD [16]. Alike to some patients after HSCT, in which GVHD does not occur shortly after cell infusion, but only after a longer time, some autoimmune diseases break out after longer periods of time – during which fetal cells might have had the time to proliferate enough to cause, channel or catalyze the disease.

#### MC and transplantation success

**Fig. 2:**
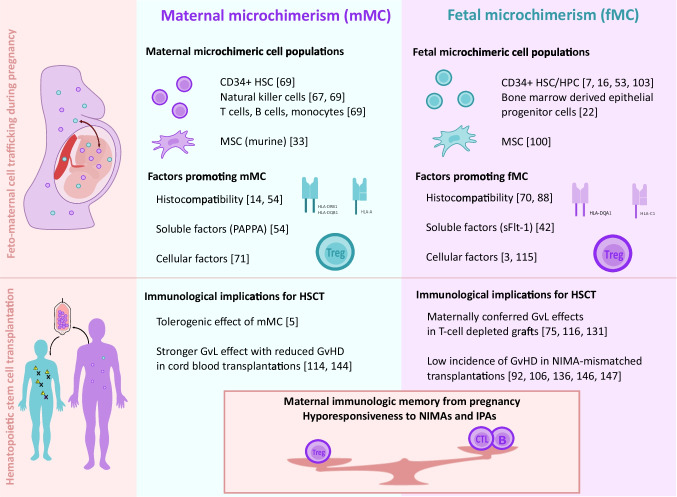
Biology and persistence of microchimeric cells and their immunological implications for hematopoietic stem cell transplantation (HSCT). The upper panel depicts maternal and fetal microchimeric cell populations and factors that positively influence their persistence. While maternal microchimeric cells are mostly reported to be hematopoietic stem cells (HSC) and mature immune cells, fetal microchimeric cells are more often detected in stem cell or progenitor populations (HPC: hematopoietic progenitor cell, MSC: mesenchymal stromal cell). Histocompatibility and the development of regulatory T cells (T_regs_) play a role in persistence of both, maternal microchimerism (mMC) and fetal microchimerism (fMC). The lower panel summarizes major implications for HSCT. An mMC (e.g., in cord blood transplantations) may confer strong graft-versus-leukemia effects (GvL) with a decreased incidence of graft-versus-host disease (GvHD). In mother-to-child transplantations, similar effects could be ascribed to fMC, and in non inherited maternal antigen (NIMA)-mismatched (sibling) transplantations low incidences of GvHD were abundantly described. Overall, an interplay between NIMA- and/or IPA-specific T_regs_, CTLs and B cells gives rise to a pregnancy- derived immunologic memory in the mother, which can be beneficial for HSCT. IPA: inherited paternal antigen, CTL: cytotoxic T lymphocyte, B: B cell

Transplantation of genetically distinct or ‘foreign’ cells from one individual to another shares similarity with implantation and development of a genetically haploidentical or ‘foreign’ fetus in the mother’s uterus. Cell transfer between mother and child can be detected as soon as six weeks post conception in virtually all mothers (-to-be) [63]. The resulting tolerated microchimerism resembles the balance between graft-versus-host and host-versus-graft reactions. Fetal hematopoietic as well as mesenchymal stem cells might engraft in maternal bone marrow during pregnancy. From there, they preserve tolerance to the semi-allogeneic fetoplacental graft [7, 16, 98]. The potent immune tolerance between mother and child confers hyporesponsiveness to NIMAs and IPAs, brought about by a unique interplay between NIMA specific T_regs_, CTLs and NIMA reactive B cells (Fig. 2). For more than twenty years, scientists and clinicians have been eager to translate the phenomenon of feto-maternal immune tolerance to donor choice or prevention of GvHD in HSCT. In 2002 Jon J. van Rood was the first to address the impact of NIMAs and NIPAs on the outcome of HSCT. In that first study comprising 269 children and adults receiving an either NIMA- or NIPA-mismatched T-cell replete haploidentical HSCT, he indeed observed less acute GvHD, and transplant related mortality (TRM) for NIMA-mismatches. Yet, there was no beneficial effect regarding graft failure rates or 2-year overall survival (OS) [141]. After that pioneering work, many others have engaged to elucidate the impact of feto-maternal immune tolerance in HSCT.

### Detection of feto-maternal chimerism before haploidentical HSCT

Haploidentical HSCTs had been performed since the late 1970ies, but were associated with increased rates of graft rejection, delayed engraftment, and GvHD [12, 38]. These adverse effects were accounted to be mainly T-cell mediated, and T-cell depletion of the graft was implemented in haploidentical HSCT before the adaptation of conditioning regimens and post-transplant immune suppression [9]. Van Rood’s pioneering study was published before the adaptations and inspired research in the field of implications of feto-maternal microchimerism in HSCT. In a few single-case reports and small cohort studies, the presence of microchimeric cells in donor and/or recipient was implemented in donor choice before T-cell replete haploidentical HSCT. The detection of microchimeric cells in donor and/or recipient was performed by HLA-nested PCR with sequence-specific primer typing (PCR-SSP) [61, 99, 100, 120, 121, 150]. The study cohorts comprised both, pediatric and adult, subjects most commonly diagnosed with advanced hematologic malignancies. Mainly, the selection of a microchimerism-positive donor was reported to be beneficial with successful engraftment, GvL effects and acceptable rates of acute and chronic GvHD, demonstrating both the feasibility and possible advantages for implementation of donor-recipient immune tolerance assays in haploidentical HSCT. Thus, first reviews by Tatsuo Ichinohe (2005) and Takanori Teshima (2006) summed up favorable effects of feto-maternal tolerance in allogeneic HSCT pleading for prospective clinical studies [60, 135]. In 2007, yet another study further emphasized implications of possible microchimeric cells by demonstrating that first-born siblings had the best outcome in regard to acute GvHD, relapse, and overall survival after HLA-identical sibling transplantation. Even though it was not assessed whether these outcomes could be accounted to NIMA-mismatching, the study still demonstrated a possible implication of transmaternal sibling microchimerism [20]. By 2010, more insight into the regulatory mechanisms determining persistence of feto-maternal microchimerism had been obtained, warranting further elucidation of regulatory and effector balances as criteria for donor selection in HSCT [59].

### Retrospective analyses of outcome regarding MC, NIMA-matching, and relationship

Table 1 summarizes a selection of publications focusing on the influence of microchimeric cells, NIMA-matching or relationship between donor and recipient on the outcome of HSCT. Utilizing T-cell depleted grafts, mother-to-child transplantations were described to be beneficial regarding better event-free survival (EFS) or OS, and less TRM, but particularly in significantly reduced risk for relapse [74, 114, 129]. This maternally conferred GvL effect without an increased risk for GvHD might be an advantageous consequence of the maternal immunologic memory derived from pregnancy. Interestingly, this effect has not been described as clear with T-cell replete grafts, where study reports are rather diverse. In a large cohort study by Wang et al., mother donors were inferior to father donors in respect to non-relapse mortality (NRM), acute GvHD, and OS [144]. Yet, sibling donors in the same study were divided into NIMA- or NIPA-mismatch groups and, as described earlier, NIMA-mismatched siblings were associated with a lower incidence of acute GvHD compared to all other donors. Two years later the group verified these results in a prospective study but could not find improved outcomes regarding chronic GvHD, TRM, relapse or survival. Interestingly, NIMA-mismatches were associated with earlier recovery of naïve T_regs_ compared to IPA-mismatches, and higher percentages of those at day 30 after transplantation resulted in lower incidence of acute GvHD grade II-IV [145]. T_regs_ can successfully counteract humoral and cellular alloresponsiveness against the fetus in mothers. Accordingly, the onset of acute GvHD is accompanied by a lack of T_regs_ that could suppress T-cell proliferation [40]. Grafts from NIMA-mismatched donors were reported to result in the lowest rates of acute GvHD grade II-IV in most studies [90, 104, 134, 144, 145]. On the contrary, for cord blood HSCT there was no benefit in NIMA-matching for the incidence of acute or chronic GvHD. Furthermore, it were NIMA-matched grafts that increased OS and decreased TRM rates [111].

**Table 1:** Transplantation success: selected references regarding influence of MC, NIMA-matching or donor relationship on the outcome of HSCT. BM: bone marrow, CML: chronic myeloid leukemia, DFS: disease free survival, EFS: event-free survival, GvHD: graft-versus-host disease, GvL: graft-versus-leukemia effect, GRFS: graft-versus-host and relapse free survival, HSCT: hematopoietic stem cell transplantation, IPA: inherited paternal antigen, LFS: leukemia free survival, MC: microchimerism, MDS: myelodysplastic syndrome, MMUD: mismatched unrelated donor, MSD: matched sibling donor, MUD: matched unrelated donor,, NHL: non Hodgkin lymphoma, NIMA: non inherited maternal antigen, NRM: non relapse mortality, OS: overall survival, PBSC: peripheral blood stem cells,, PFS: progression free survival, ptCy: post transplantation cyclophosphamide; SAA: severe anaplastic anemia, TRM: transplant related mortality, UCBT: umbilical cord blood transplantation

Reference/ Year	Study size			Diseases	HSCT/ Donor	Graft source/ Manipulation	Serotherapy	ptCy	Major findings regarding MC/NIMA/kinship	MC/NIMA outcome
	Center	Subjects	*n*							
Stern et al. 2008 [129]	Two center	Pediatric	118	Acute leukemias	Haplo Parents	PBSC T cell deplete	No	No	* ↑ 5-year EFS in mother-to-child with ↓ risk for relapse and TRM * No significant influence on rejection rates or acute GvHD	↑
Rocha et al. 2012 [111]	Multi center	Pediatric + adult	164	Hematologic	MMUD (single mismatch)	Cord blood	No	No	* ↑ OS and ↓ TRM in NIMA-matched compared to NIMA-mismatched UCBT * ↓acute and chronic GvHD after UCBT compared to haplo HSCT, no benefit in NIMA-matching	↑
Dobbelstein et al. 2013 [31]	Multi center	Pediatric + adult	11,365	Hematologic	MSD	BM, PBSC T cell replete (95%)	No (excluded)	No	* ↓ acute GvHD grade II-IV and chronic GvHD when donors younger than recipients * No overall positive effect on incidence of relapse or OS	n.d.
Wang et al. 2014 [144]	Single center	Pediatric + adult	1210	Hematologic	Haplo Parents, sibling, child	BM, PBSC T cell replete	Yes	No	* Father donors associated with ↓ NRM, acute GvHD, and ↑ OS compared to mothers * NIMA-mismatched sibling donors associated with ↓ incidence of GvHD compared to all other donors	(↑)
Kruchen et al. 2015 [74]	Single center	Pediatric	10	Diverse	Haplo Parents	PBSC T cell deplete	Yes	No	* ↑ 3-year OS when MC^+^ mother donor compared to MC^−^ mother or father * ↓ 3-year incidence of relapse when MC^+^ mother donor in leukemia patients	↑
Sun et al. 2015 [132]	Single center	Pediatric	111	Acute leukemias	Haplo Parents	Combined BM and PBSC Unmanipulated	Yes (most)	No	* Best 5-year OS after father-to-son HSCT, followed by mother-to-daughter HSCT * Worst 5-year OS after father-to-daughter HSCT	↓
Berger et al. 2016 [13]	Multi center	Pediatric	33	Hematologic	Haplo Parents, sibling	Mainly BM T cell replete	No	Yes	* ↓ risk of relapse when mother donor, female donor or female patient	↑
Inamoto et al. 2016 [62]	Multi center	Pediatric + adult	23,302	Hematologic	MSD vs. MUD	BM, PBSC, and CB n.d.	Some	No	* ↑ GRFS in female patients * Worst outcome in female-to-male HSCT compared to all other variations	↓
Wang et al. 2016 [145]	Single center	Pediatric + adult	57 + 88	Hematologic	Haplo Sibling	Blood and marrow Unmanipulated	Yes	No	* ↓ acute GvHD grade II-IV after NIMA-mismatched compared to NIPA-mismatched HSCT * No effects of NIMA-mismatching on chronic GvHD, TRM, relapse, DFS or OS	↑
Bertaina et al. 2018 [15]	Multi center	Pediatric	343	Acute leukemias	Haplo (parents) vs. MUD vs. MMUD	BM, PBSC αβT/CD19-depletion (haplo)	Yes	No	* ↓↓ acute GvHD grade II-IV after αβ/CD19-depletion HSCT * TRM and GRFS similar after αβT/CD19-depletion and MUD HSCT	n.d.
Ma et al. 2019 [81]	Single center	Pediatric + adult	92	Leukemias	Haplo (mother) vs. MUD	BM, PBSC Unmanipulated	Yes	No	* ↓ 2-year incidence of relapse in mother group; 2-year OS and LFS comparable * ↑ acute GvHD grade II-IV in mother group, especially in mother-to-son HSCT	Diverse
Pingel et al. 2019 [104]	Multi center	Adult	445	Acute leukemias	MMUD	BM and PBSC T cell replete	(Yes) approx. half	No	* No significant differences between NIMA- and NIPA-mismatched grafts * Very low frequency of NIMA-matches in NHL, CML, and MDS patients	No difference
Xu et al. 2019 [148]	Multi center	Pediatric + adult	392	SAA	Haplo Parents, sibling	BM, PBSC T cell replete	Yes	No	* No significant differences between father, mother, child or sibling donors * ↑ chronic GvHD when mother is donor compared to all other donors	↓
Mariotti et al. 2020 [84]	Multi center	Pediatric + adult	990	Hematologic	Haplo 1st or 2nd degree relative	BM, PBSC Unmanipulated	No	Yes	* ↓ OS and PFS when mother is donor compared to father	↓
Rocha et al. 2021 [110]	Multi center	Pediatric	144	Acute leukemias	Haplo Parents, sibling	BM, PBSC T cell replete	No	Yes	* ↑ risk of chronic GvHD when mother is donor * ↓ OS and GFRS when mother is donor	↓
Tang et al. 2021 [134]	Single center	Pediatric + adult	378	Hematologic	Haplo Sibling	BM, PBSC Unmanipulated	Yes	No	* ↓ rate of acute GvHD grade II-IV in NIMA-mismatched HSCT * ↑ OS and DFS in NIMA-mismatched HSCT	↑
Ruggeri et al. 2022 [114]	Multi center	Pediatric + adult	409	Acute leukemias	Haplo Mother, other related	Mostly PBSC, less BM Mostly es vivo T cell depleted	Yes	No	* ↓ relapse in mother-to-child T cell depleted HSCT * Maternal immunologic memory exerts GvL responses	↑
de Lima et al. 2022 [29]	Two center	Adult	103	Hematologic	Haplo Mother, ‘other donors’	BM, PBSC T cell replete	No	Yes	* Mother donor risk factor for OS, PFS, and relapse	↓
Mehta et al. 2023 [90]	Single center	Adult	412	Hematologic	Haplo Parents, sibling	BM, PBSC T cell replete	No	Yes	* ↓ risk for acute GvHD grade II-IV in NIMA-mismatched HSCT * No significant difference in outcome between daughters and sons (no NIMA-/ IPA-matching applied)	↑
Pruitt et al. 2023 [107]	Single center	Adult	299	Mostly acute leukemias	Haplo Parents, sibling, child, other	PBSC	No	Yes	* No differences in any outcome based on donor-recipient relationships	No difference

### Cord blood transplantation

As described above, maternal microchimerism is established in all offspring as fetal microchimerism is in all mothers. Umbilical cord blood (CB) is yet another source of HSCs for transplantation in various diseases and associated with some benefits including lower incidence of acute and chronic GvHD compared to bone marrow in HLA-identical sibling transplantation [112]. Reduced GvHD was even observed when the GvL effect was strengthened. In a group of patients with minimal residual disease (MRD) at the time of transplantation, the probability of leukemia relapse was lower in the group receiving a CB transplant compared to the groups receiving either HLA-matched or –mismatched grafts [92]. An explanation for this phenomenon is lacking, but might be found in circulating maternal cells in the CB graft. Van Rood et al. could show that leukemia patients, who shared one or more HLA-A, -B, or –DRB1 antigen with their CB donor’s IPAs, had a significant decrease in the incidence of leukemic relapse post transplantation. The benefit in GvL was not accompanied by an increase in GvHD [142]. These findings led to the hypothesis that maternal microchimeric cells that have been exposed to IPAs during pregnancy might be able to recognize a broader range of antigens in leukemic blasts after transplant [66]. Many CB banks introduced HLA typing of the CB donor’s mothers to characterize NIMAs. Inclusion of NIMA-matching in CB transplantation might increase the probability of finding the most effective CB unit and allows for HLA-mismatches that can be counterbalanced by NIMA-matches [37, 75, 106, 137].

### Retrospective analyses of outcome regarding MC and relationship in regimens with post-HSCT immune suppression

Post-transplantation immune suppression with cyclophosphamide (ptCy) was designed to target and deplete alloreactive T cells a few days after transplantation. Alloreactive host and donor T cells are activated and hence proliferate in the first days [89]. Cyclophosphamide only eliminates proliferating T cells reprieving the non-reactive ones. A clonal elimination of alloreactive T cells could be described [49, 82]. Application of ptCy following haploidentical HSCT achieved results comparable to HLA-matched transplantations with low rates of acute and chronic GvHD as well as low NRM [11]. It might be speculated whether the treatment with ptCy fully eradicates beneficial maternal T cells or deprives them of their memory. The mechanism of destroying alloreactive T cells might also exactly resemble what maternal T_regs_ would accomplish and the beneficial effects ascribed to NIMA-mismatching might simply be overrun. The results obtained in studies employing ptCy and not matching for NIMA showed generally worse outcomes, especially regarding OS, and progression-free survival (PFS) for mother-to-child transplantations compared to father donors and other donors [29, 84, 110]. One of the groups declared mother donors as risk factor for OS, PFS, and relapse [29]. Only in one smaller cohort the risk for relapse was reduced when the mother donated bone marrow for her child [13]. These findings held also true for female donors and female recipients in general in that analysis [13].

### NIMA-matching and immune reconstitution

Early recovery of naïve T_regs_ was associated with NIMA-mismatching and improved outcome after HSCT. Four weeks after transplantation, absolute cell counts of T_regs_ in general were higher after NIMA-mismatched compared to NIPA-mismatched HSCTs [145]. Although it had been postulated that NIMA-mismatching benefits T-cell immune reconstitution [135], Wang et el. disconfirmed this statement by showing similar reconstitution rates of CD3^+^ T cells, CD4^+^ T cells, and CD8^+^ T cells after NIMA- or NIPA- mismatched HSCT [145]. The same held true for CD19^+^ B cells, while data on NK cell reconstitution is missing.

Whereas T cells are highly affected by treatments like ptCy, NK cells are spared. Two of the depicted studies also analyzed KIR ligand mismatches in their cohorts. Stern et al. reported a trend towards more KIR ligand mismatches in mothers resulting in a superior outcome in mother-to-child transplantations [129]. Berger et al. also described a trend for KIR alloreactivity towards improved survival rates, lower risk for relapse, and less TRM. Risk for relapse was also associated with female sex in general, for both donor and recipient [13]. Furthermore, in a preclinical study, it was shown that NK cells from mothers harboring fetal microchimeric cells have a higher degranulation potential towards their child’s leukemic blasts in vitro [86].

Taken together, differences in transplantation protocols hamper the comparison of larger cohorts as well as observations over a longer period of time, and might thus conceal the true impact of fetal microchimerism for GvL effects in leukemia patients. There is also a clear urgency for direct proof of a persisting microchimerism or analysis of NIMA- and IPA-matching. For further elucidation, preclinical studies remain the best-suited approach in deciphering the feto-maternal immune tolerance benefit for HSCT.

## Further lessons from solid organ transplantation, preclinical studies, and mouse models

### Feto-maternal microchimerism in solid organ transplantation

The concept of establishing microchimerism by (co-)implanting immune cells during solid organ transplantation is well accepted. Yet, solid organ transplantation might also be affected by natural microchimerism. T cells primed against IPAs present on fetal microchimeric cells in the mother have been reported as potentially aggravating solid organ transplantation for the mother [138]. Conversely, the presence of maternal microchimerism or a shared HLA-DR identity with their mother was strongly associated with impressively decreased, even abolished, cellular rejection of maternal liver transplants in pediatric recipients [149].

Burlingham et al. showed already in 1998 in a retrospective study that in kidney transplantation from one sibling to another, NIMA- but not NIPA-matching resulted in better graft survival five and ten years after organ transplantation [21]. Contradictory, in the same cohort, NIMA-matched grafts demonstrated an increased number of early rejection, which could be counteracted by donor blood transfusion prior to organ transplantation. Most subjects of the cohort were treated before introduction of new immunosuppressive drugs around 1986 [21]. A more recent retrospective study on kidney transplantations from a parent to a child included subjects transplanted between the years 1985 and 2019. Here, the results were dependent on the children’s age at the time point of transplantation. In younger children, aged one to four years, transplantation of a kidney from their mothers was associated with less treatment for rejection compared to children receiving kidneys from their fathers [36]. In kidney transplantation, the concept of NIMA-associated ‘split tolerance’ has been discussed, conflating the observation of higher early rejection rates and increased long term graft survival. Therapeutically, addressing acute rejection is more feasible compared to long term graft rejection [78].

In a murine model of heart transplantation, transplant rejection was only prevented in mice that harbored maternal microchimeric cells and supporting T_regs_. T_regs_ migrated to the transplant and secreted IL-10 and tumor growth factor (TGF-) β thereby impeding acute rejection. MMC-negative mice also lacked the supporting T_regs_ and were not able to protect the graft allowing for graft rejection [32].

### *In utero* hematopoietic cell transplantation

In a process called *in utero* hematopoietic cell transplantation (IUHCT), allogeneic hematopoietic stem cells are being transfused *in utero* into the developing fetus. This procedure is supposed to outperform normal HSCT in terms of less rejection and requiring less immunosuppressive therapy, but is foremost confronted with low engraftment rates. The potentially negative influence of maternal microchimeric cells on donor chimerism is currently being analyzed and discussed. By breeding T-cell deficient female BALB/c mice to wild-type BALB/c males it could be demonstrated that lack of maternal T cells in the offspring led to improved engraftment of allogeneic donor cells (B6). Maternal conventional T cells appeared to be a driving force of graft rejection. This hypothesis was even strengthened when it could be demonstrated that MHC matching between mother and graft increased engraftment in MHC-mismatched fetuses and led to engraftment rates comparable to MHC-matched grafts [97]. Kandasamy et al. [68] recently demonstrated the engagement of maternal dendritic cells (DCs) in the described immunogenic responses. DC-deficient and DC-replete mice received either paternal or maternal-derived haploidentical or fully allogeneic IUTs. DC depletion showed no direct impact on donor cell chimerism, but instigated expansion of T_regs_ and increased cytokine expression associated with immune inhibition like IL-5, IL-6, IL-10, and TGFβ [68].

Besides whole cells, MHC-bearing exosomes offer a possibility to analyze alloreactivity responses. In a murine model, *in utero* infusion of allogeneic MHC exosomes resulted in a diminished immune response of the recipient’s lymphocytes against alloantigens. The in vitro alleviated immune response did not translate into prevention of rejection of a postnatally transplanted allogeneic skin [24]. Bracamonte-Baran et al. [19] showed that maternal microchimeric cells in mice produce extracellular vesicles (EV) which bear NIMAs. These NIMA-EVs can be taken up by the offspring’s dendritic cells resulting in presentation of maternal MHC class I and II molecules, a so called “cross decoration” [19]. Intrigued by these findings in mice, a recent study could adopt this process to humans. A small percentage, around 6%, of fetal dendritic cells in cord blood was decorated with NIMAs. More than half of NIMA-bearing DCs expressed programmed cell death ligand-1 (PD-L1), compared to less than 20% of regular DCs. NIMA and PD-L1 expressing EVs were also reported in cord blood [78].

Altogether, translation of the principles of feto-maternal immune tolerance into clinical application remains challenging. Introduction of elegant murine models and development of new in vitro analyses of specific alloreactivity responses are indispensable to further elucidate mechanisms underlying phenomena like split tolerance.

## Concluding remarks

The elucidation of the biology and the implications of the immune tolerance between mother and child remains a topic of great interest. For more than two decades, research has been ongoing to translate the observed immune tolerance mechanisms into improving outcomes after HSCT. Pioneering studies emphasized the role for NIMA-mismatching in selection of sibling, child, or maternal donors. In T-cell deplete haploidentical HSCT, transplantations from mother to child appealed with low risk for relapse in line with no increased risk for GvHD. For T-cell replete haploidentical HSCT, results were not so clear, yet NIMA-mismatched sibling transplantations were associated with low incidences of GvHD. Today, graft manipulation and employment of new, more specific and powerful immunosuppressive drugs and strategies improved the safety and outcome after HSCT. Beneficial effects ascribed to NIMA-mismatching, like reactive maternal T cells targeting malignant cells in the child, might simply be abolished by stronger immune suppression. Nevertheless, preclinical studies still attest the potent role of microchimeric cells not only for HSCT, but also for solid organ transplantation, and cord blood banks started to include NIMA-matching in donor selection processes. The recent perception of a microchimeric stem cell sanctuary that harbors only one dominant microchimeric cell population, which will be exchanged with every pregnancy, would leave the hypothesis that mothers are only optimal donors for their lastborn child, while sons would always present a suitable donor for their mother. To answer these questions, further studies are needed that address the specific detection and decipher the biological features of microchimeric cells in donor and recipient, which has become feasible with new technologies like single-cell OMICS analyses.

## Data Availability

No original data was generated for this review article.
